# A Randomized Clinical Trial Comparing Implants Placed in Two Different Biomaterials Used for Maxillary Sinus Augmentation

**DOI:** 10.3390/ma16031220

**Published:** 2023-01-31

**Authors:** Francisco Correia, Sónia Alexandre Gouveia, Daniel Humberto Pozza, António Campos Felino, Ricardo Faria-Almeida

**Affiliations:** 1 Department of Oral Surgery and Periodontology, Faculty of Dental Medicine, University of Porto, 4200-393 Porto, Portugal; 2 Intelligent Systems Associate Laboratory (LASI), Department of Electronics, Telecommunications and Informatics (DETI), Institute of Electronics and Informatics Engineering of Aveiro (IEETA), University of Aveiro, 3810-193 Aveiro, Portugal; 3 Department of Biomedicine, Faculty of Medicine, University of Porto, 4200-319 Porto, Portugal; 4 Department of Histology, Faculty of Nutrition and Food Sciences, University of Porto, 4150-177 Porto, Portugal; 5 Institute for Research and Innovation in Health and IBMC, University of Porto, 4200-135 Porto, Portugal

**Keywords:** biomaterials, dental implants, autologous bone, porcine xenograft, bone regeneration, ceramic prosthesis

## Abstract

The objective of this study was to compare marginal bone loss, surgical and clinical complications, and dental implant survival rate in bilateral maxillary sinus augmented by autologous or porcine xenograft. A randomized controlled clinical trial using split-mouth design enrolled 12 consent adult patients (59.7 ± 8.7 years), who received bilateral maxillary sinus floor augmentation for oral rehabilitation with implant-supported prosthesis. Each patient received both the autologous bone from the mandible (control) or porcine xenograft (test) during the random bilateral sinus lift surgery. A total of 39 dental implants were placed in the posterior maxilla of the 12 patients after 6 months, being rehabilitated after the respective osseointegration period. Both graft materials demonstrated a high implant survival rate at 12 months: 95% for the xenograft side, only 1 implant without osseointegration, and 100% for the autologous side. Radiographic bone loss was low and similar for both groups: control group with a mean of 0.063 ± 0.126, and test group with a mean of 0.092 ± 0.163. No major surgical-related complications have occurred. Only one patient had several prosthetic complications due to fractures of prosthetic components. The maxillary sinus augmentation procedure, both with autologous bone and porcine xenograft materials, is an excellent clinical option procedure for the prosthetic rehabilitation of atrophic maxillae, with low marginal bone loss after one year follow-up, few clinical complications, and a high implant survival rate.

## 1. Introduction

The posterior maxilla represents a challenge for oral rehabilitation with dental implants since bone height is usually around 4 mm, or even less in approximately 43% of the patients [1]. The limited availability of alveolar bone for dental implant placement can result in a lack of primary stability and it is difficult to achieve osseointegration. Furthermore, the reduced alveolar bone will generate hygiene-related difficulties and functional problems. For these reasons, bone augmentation before surgery is mandatory to allow proper implant-supported prosthesis rehabilitation [2,3]. To support this idea, a retrospective study estimated that an elevation of the maxillary sinus will be required in 54.4% of the cases where implants were planned to be placed in the posterior maxillae [4].

In this context, several surgical techniques are currently described in the literature to allow the placement of dental implants. Before or at the same time of implant placement, maxillary sinus augmentation can be performed by lateral osteotomy or by maxillary sinus elevation with osteotomes or osseodensification drills [5,6]. Short, ultra-short, and zygomatic implants are also options to rehabilitate an atrophic maxilla [5,7,8]. When available bone measures are less than 5 mm in height, lateral sinus lift with bone graft is the preferred therapeutic option to allow implant placement [1,2,9,10,11]. Treatment choice should be based primarily on anatomy, sinus pathology, assessment of the extent of desired bone augmentation, preoperative alveolar bone size, and bucco-palatal sinus dimension. It is also important to consider other patient-related factors, including general health status, smoking habit, oral hygiene, and patient preferences [1,2,5].

Different biomaterials, including autologous bone graft (gold standard), freeze-dried bone allograft, lyophilised demineralised frozen allograft, deproteinised freeze-dried bone allograft, porcine or bovine xenografts; and different alloplastic materials such as beta-tricalcium phosphate, calcium sulphate, hydroxyapatite, bioactive glass, calcium carbonate, polyglycolic-polylactic are available to be used as bone grafts in lateral sinus lift procedures [8,12,13]. The different biomaterials can be applied alone or in combination with each other, including autologous bone, hydroxyapatite, hyaluronic acid, collagen, tricalcium phosphate, platelet rich fibrin, platelet-derived growth factor, platelet-rich plasma (PRP), mandibular periosteum-derived cells, human bone morphogenetic protein-7 recombinant, bone marrow derived mesenchymal stem cells, concentrated growth factors, mesenchymal stem cells, among others [8,12,13,14,15,16,17,18]. Regarding the success rate of implants placed in regenerated posterior maxillae, it is possible to find cumulative survival rates close to 100% [7,19]. Nevertheless, the analysis of survival rate must take into consideration several factors including complications as the Schneider membrane perforation [20], chronic sinus infection, and the patient history of periodontal disease as well as the development of periimplantitis [7].

The use of porcine xenograft is reported in the literature as a good alternative to autologous bone graft for sinus lift [21]. A previous study of Barone A. et al. 2005 [22] tested autologous bone in comparison to 50% autogenous bone and 50% corticocancellous porcine xenograft with good results. The authors raised the question if it was possible to obtain the same results with a minimal amount or no autologous bone in the mixture.

This randomised split-mouth controlled clinical trial aimed to compare a xenograft with 10% collagen biomaterial with autologous bone for maxillary sinus augmentation in terms of marginal bone loss, surgical and clinical complications, and dental implants survival rate after a 12-month prosthetic rehabilitation period.

## 2. Materials and Methods

### 2.1. Study Design

This randomised clinical trial (RCT) followed the CONSORT Statement and World Medical Association Declaration of Helsinki Guidelines [21]. This study was approved by the Ethics and Research Committee of the Faculty of Dentistry, University of Porto, Portugal, with number 00977 and registered on trial.gov (NCT01836744).

Twelve consecutive patients were included in this study that was performed at the Faculty of Dentistry, University of Porto, Portugal. A total of 24 maxillary sinuses were divided into two groups (autologous graft or xenograft). Additional information can be found in our previous paper [22]. Software randomisation was used to select the graft material (autologous graft or xenograft) to be used in each regenerated sinus (www.randomizer.org). The result of the randomised assignment was placed in a sealed opaque envelope and concealed unit and opened by the surgeon only once both sinus Schneider membranes were raised, ensuring the allocation and groups were balanced. The allocation scheme was kept confidential from clinical examiners and patients.

Adult patients enrolled in this RCT understood and signed the informed consent; had previous bilateral posterior maxillary sinus floor augmentation; and had no serious oral or systemic untreated diseases. Additionally, the oral health status of patients was checked in the first consultation, including the previous dental history. Before starting the oral rehabilitation all the patients received proper care, whenever needed, including teeth restoration, periodontal treatment, and occlusal adjustments to allow a heathy oral environment for dental implants treatment.

### 2.2. Treatment Procedures

The surgical procedure for sinus lift augmentation is described in our previous paper [22] and comprises dental polishing, antibiotics, and mouthwash before surgery. All surgeries were performed according to the technique described previously [9], which is comparable in time and duration. The osteotomy was performed with a piezoelectric instrument (NSK VarioSurg™, Tokyo, Japan) or with a spherical diamond tip (NSK™, Tokyo, Japan). The bilateral Schneider’s membrane was carefully elevated for the insertion of either porcine xenograft Osteobiol mp3^®^ (Tecnoss™, Torino, Italy), granulometry between 600–1000 µm with 10% collagen of type I and III) [23,24]; or intraoral autologous bone graft. The amount of graft used in each sinus was individualised according to anatomical characteristics and the amount of bone height required for implant placement during the treatment plan. A collagen membrane (Osteobiol, Tecnoss™, Torino, Italy) covered the lateral osteotomy. Polyamide 4.0 (Supramida™, B Braun, Melsungen, Germany) was used to suture the flaps. Six months after first surgical intervention, the same surgeon performed the second surgical phase administrating local anaesthesia. After the full-thickness incision, the flaps were elevated. Submerged dental implants (OsseoSpeed TX™, Astra Tech Implant System, Dentsply Sirona Implants, Möndal, Sweden) were placed following the manufacturer’s recommendations.

Six months after implant placement, the second surgical phase was performed placing the healing abutments in the cases of single crowns, or UniAbutments 3.5/4.0 (Astra Tech Implant System, Dentsply Sirona Implants, Mölndal, Sweden) with a Heal Cap for bridged rehabilitations with 2 to 3 implants. Individually adjusted screwed metallic-ceramic crowns with a TiDesign 3.5/4.0 abutment (Astra Tech Implant System, Dentsply Sirona Implants, Mölndal, Sweden) or screwed metallic-ceramic bridges were used in all implants and no connection tooth implant was performed. All patients received the same prosthesis design (single or bridge), which was manufactured by the same prosthetics specialist, fitting, and adjusted in the same manner by the same clinician.

### 2.3. Follow-Up

Follow-up was every 6 months after prosthetic loading and included individualised professional maintenance, removal of prostheses for clinical evaluation, and adjustment of occlusion if necessary.

Clinical and radiographic evaluations, including implant stability, marginal bone loss, prosthetic or biological complications, and patient preferences, were performed one year after implant loading. The implant success rate was based on osseointegration previous or after loading. Implant mobility and/or any infection dictating implant removal was considered a failure. Prosthesis failure was considered whenever planned oral rehabilitation was not possible.

Changes in peri-implant marginal bone level were evaluated by periapical radiographs taken with the paralleling technique at initial prosthetic loading and after one year of implant function. Mesial and distal measurements were performed between marginal bone level and implant/abutment junction. Changes in bone level in single implants were averaged at the patient level and then at the group level. The marginal bone levels of the periimplant were measured using Scion Image software (Scion Corporation, Frederick, MD, USA). The software was calibrated for each image using the respective implant length. Mesial and distal bone crest level measurements, adjacent to each implant, were made with an accuracy of 0.01 mm. The reference points for the linear measurements were the coronal margin of the implant collar and the most coronal point of bone-implant contact [25].

### 2.4. Statistical Analysis

Data organization and descriptive statistics were performed in Microsoft ExcelTM16.10. Statistical analysis and visualisation were performed using the Estimation Stats web application (https://www.estimationstats.com/, [26] (accessed on 3 January 2022)). For each comparison, the effect size (mean paired differences), the 95% confidence interval (CI) and the *p*-value (probability of obtaining a mean difference at least as extreme as that observed in this study) were reported as effect size [CI lower bound, CI upper bound] (*p*-value). Each CI is a bootstrap bias-corrected and accelerated (non-symmetric) interval and each *p*-value is the result of a permutation test, based on 5000 bootstrap samples, obtained by resampling with replacement of the original data. The results were presented on a table format and displayed in a Gardner–Altman plot, showing the original data, sampling distributions (distribution of the mean difference under the null hypothesis of no effect) and CIs obtained via the bootstrap approach. Conventional parametric and nonparametric statistical testing (5% significance level) was also conducted to corroborate the results of the bootstrapped analysis. This was accomplished with IBMTM SPSSTM (25.0, IBM Corp., Armonk, NY, USA). The comparisons were based on paired t-test (parametric) and Wilcoxon signed-rank test (non-parametric), with the normality assumption being tested via the Shapiro–Wilk test.

## 3. Results

### 3.1. Patient and Intervention Characteristics

The sample of this randomized clinical trial with a split-mouth design included 12 patients (6 males and 6 females), 24 sinus floor augmentations and 39 dental implants. The average age of the patients was 59.7 ± 8.7 years old, 6 patients were non-smokers, 3 light smokers, 1 heavy smoker and 2 former smokers. The great majority of the sample (9 out of 12) presented at least one pathology and was intaking at least one prescribed medication (11 out of 12). Autologous bone was harvested from the mandible branch in 83.3% of the patients and from the chin in 16.7% of the cases.

For the surgical intervention, one patient reported preferring the xenograft approach over the side that received autologous bone. After one year of function, patients were asked about their treatment preferences and reported that ‘Neither’ or ‘Both procedures were equally good’, demonstrating that there was no preference for one of the sinus lift procedures.

### 3.2. Implant Survival

**Figure 1:**
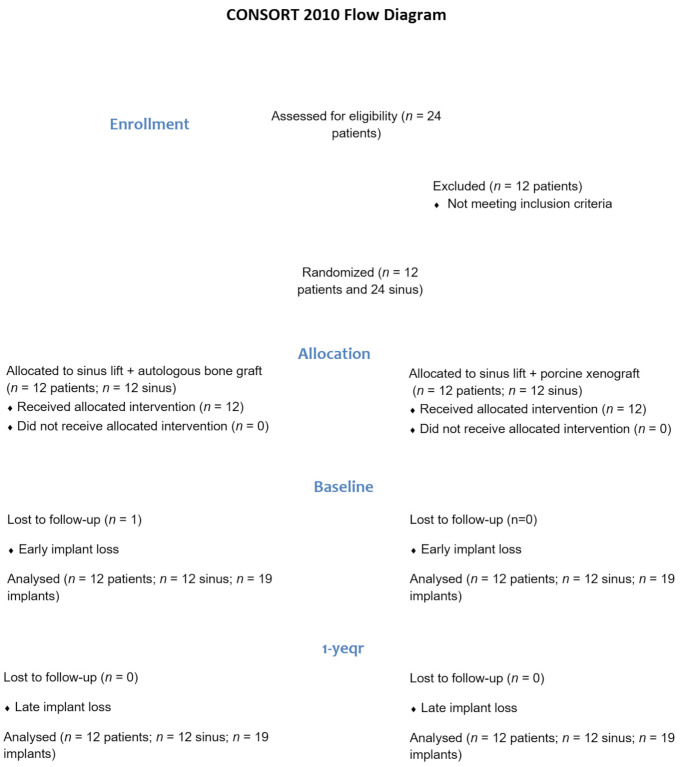
Flow Diagram.

The flow diagram in Figure 1 outlines the entire process concerning enrolment and allocation of patients, as well as the evaluation of the results at the baseline (beginning of the trial) and after one year of follow-up. Overall, 24 patients were invited to be included in the study, but only 12 met the inclusion criteria. Each of the 12 patients included received the sinus lift by xenograft or autologous bone, randomized for each side. A total of 39 implants were placed in augmented maxillary sinuses: 16 implants with 9 mm of length and 23 with 11 mm of length. All implants had a platform of 4.0 mm. However, 38 implants were evaluated since one of the implants (9 mm of length) at the xenograft side did not osseointegrate. There was no need to replace this implant as its absence compromised neither the prosthesis confection nor the patient’s masticatory function. The implant survival rate, after one year of follow-up, was 95% for the xenograft side and 100% for the autologous graft side.

### 3.3. Survival of the Prosthesis and Hardware Complications

Of the 12 patients enrolled in this study, 2 were rehabilitated with a single crown on each augmented side and 10 rehabilitated with splinted crowns or bridge crowns. Regarding prosthesis complications, there was only one patient experiencing multiple prosthetic complications: fractures in both right and left ceramic bridges, eight screw fractures and a multi-unit fracture in each side.

### 3.4. Periimplant Soft Tissue Condition and Biological Complication

**Figure 2:**
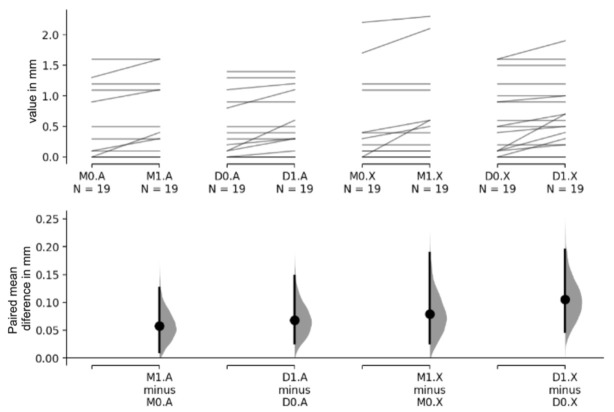
Gardner–Altman plots showing the observed bone loss values for all subjects. The upper axes display the data where each paired set of observations (for the same subject) is connected by a line. In the lower axes, the bootstrapped paired mean difference between the evaluation moments t1 and t0 is plotted as a sampling distribution (grey). Mean differences are depicted as dots and 95% CIs are indicated by the ends of the vertical bars. Legend: M—mesial, D—distal, A—autologous bone graft, X—porcine xenograft, 0—t0, and 1—t1, N—number of implants per group, mm—millimetres.

The mean radiographic bone loss one year (t1) after prosthesis delivery (t0) was 0.068 ± 0.144 mm in mesial and 0.087 ± 0.149 mm in distal (Figure 2). It is possible to verify that most implants presented stability in bone levels for the 12-month follow-up period (represented by the flat lines). However, some implants had an expected small increase in bone loss, represented by the lines with a positive inclination. Implants placed in both biomaterials presented similarly low levels of bone loss.

Table 1 shows the results of bone loss according to the biomaterial used (autologous graft versus xenograft) and the moment of evaluation (t0 and t1), aggregating the mesial and distal measurements. The results show a statistically significant bone loss both for the autologous graft (0.063 ± 0.126 mm, *p* = 0.004) and for the xenograft group (0.092 ± 0.163 mm, *p* = 0.001), with no significant differences between materials (*p* = 0.390). Table 2 presents the same analysis (t0 and t1), distinguishing the mesial and distal locations to identify any differences concerning location. Regarding the bone loss associated with the autologous graft, the paired difference between temporal evaluations was 0.0579 mm (0.0105–0.126 mm, *p* = 0.129) for the mesial location and 0.0684 mm (0.0263–0.147 mm, *p* < 0.001) for the distal location. For the xenograft, the corresponding results were 0.0789 mm (0.0263–0.189 mm, *p* < 0.001) and 0.105 mm (0.047–0.195 mm, *p* = 0.008), respectively, for the mesial and distal locations. In all cases, as the 95% confidence intervals do not include the zero difference, then one can conclude that the expected paired differences are statistically significant for all cases, at 5% significance level. On the other hand, the statistical comparisons based on the *p*-value identify differences in all cases, except for mesial location and autologous material. Recalling that this effect size is measured in mm units, this constitutes a quite small value of bone loss differences for all cases, which are likely to have no clinical relevance, although being statistically significant.

## 4. Discussion

This randomised controlled clinical trial demonstrated a high survival rate for implants placed either in autologous bone grafts or in porcine xenografts after 12 months of bilateral sinus lift procedures. The clinical and radiological analysis, as well as prosthesis survival and patient preferences, were also equivalent between the groups.

The present trial studied the porcine xenograft for bone regeneration as an alternative to the autologous bone graft. Autologous bone is still considered the gold standard by several authors [23,27,28,29,30]. However, other biomaterials such as xenografts are preferred due to the morbidity associated with the autologous bone harvesting procedure, including possible postoperative complications, increased surgical time, and limited amount of autologous material, mainly for rehabilitation of large maxillary sinuses [8,28,29]. In the present study only one maxillary sinus per patient was filled with autologous bone and the opposite sinus with xenograft. There were no major differences in terms of surgical procedures or related complications, however it was easier and faster to work with the xenograft biomaterial. No other clinical complications such as displacement of the dental implant in the sinus, ostium obstruction, oral fistula, sinusitis, or bone infection that would require a transnasal or transoral approach have been observed [31]. Additionally, one patient reported preferring the xenograft approach over the side that received autologous bone after sinus surgery. However, one year later, when the interview was repeated, the same patient reported no preference. Therefore, both materials and surgical procedures can generally be considered very well tolerated, as previously described [13].

The present results demonstrated 95% success rate for implants placed and loaded in porcine xenografts and 100% for implants in the autologous bone graft group. Other studies using the same graft materials reported similar results with success rates that also varied from 95% to 100% [24,32]. In addition, previous studies and reviews of other grafts for maxillary sinus grafting (e.g., demineralised bovine bone—DBB, extraoral autologous bone graft—autologous iliac crest blocks, Beta-Tricalcium Phosphate) or mixtures of materials (e.g., autologous bone + PRP, venous blood + autologous bone, xenograft + mesenchymal stem cells, xenograft + autogenous bone), reported similar or slightly worse survival rates, varying between 83.5% up to 100.0% [8,13,18,33,34]. Nevertheless, the reported results are very positive and close to those obtained in implants placed in pristine bone. Several factors can influence the maintenance of marginal bone and osseointegration, including smoking habits, bone nature, residual bone height, biomechanical factors, timing of placement, and previous history of periodontitis [35,36,37,38,39,40,41]. In our study only one implant on the xenograft side did not osseointegrate, being in accordance with the European Association for Osseointegration consensus: longer dental implants placed in the augmented sinus, compared to implants placed in native bone, have an increased failure rate of up to 17% within 3 years [19]. The dental implants of the present study were inserted into preparations made using conventional drilling techniques. A recent study [42] demonstrated that other good alternatives, for posterior regions of the maxilla, such as piezoelectric tips or with osseodensification drills also presents excellent implant stability quotients and survival rates.

The group using xenograft biomaterial presented slightly higher mean bone loss that was considered not clinically significant at the evaluated period. The duration of follow-up is important for the bone loss evaluation, being expected that the bone loss will increase over time. For the first year, less than one millimetre of marginal bone loss is expected; however, this value can be more than two millimetres after 3 years of implant loading [43]. Previous studies with the same Osteobiol mp3 (Tecnoss™, Torino, Italy) xenograft used in our clinical trial demonstrated similar bone loss (0.2 ± 0.8 mm or 0.2 ± 0.3 mm) after one year [24]. Another study that used the same implant brand, inserted simultaneously to the lateral sinus lift procedure (xenograft + autologous bone) reported a similar average marginal bone loss of 0.27 mm for the implant one year after loading. The mean bone loss was also evaluated after 3 years of follow-up and was 0.45 mm [44]. These are positive values for porcine xenograft, allowing to predict a good longevity of the present treatment.

The marginal bone loss evaluated in our study is also comparable with that reported in previous studies using other biomaterials for sinus floor augmentation: 0.47 ± 0.31 mm for xenograft + mesenchymal stem cells and 0.41 ± 0.25 mm for xenograft + autogenous bone [35]; and 0.93 ± 0.40 mm for DBB [45]. Furthermore, the marginal bone loss obtained in the present RCT was found to be even smaller than in implants placed in sinuses augmented with DBB + autologous graft (1.06 ± 0.61 mm) or for autologous graft alone (1.19 ± 0.82 mm) [32]. Additionally, it was suggested that, in the augmented sinuses, the load distribution and the implant marginal bone loss might be related to the graft characteristics [46]. Thus, it is very important to follow up the implant’s behaviour, mainly those inserted in grafted biomaterials. Differences in manufacturing processes can cause modifications in the physicochemical characteristics of the surface of the biomaterial, leading to different biologic responses, even for grafts of the same origin [47].

It was possible to verify that even in the cases of a membrane perforation, neither the implant success rate decreased, nor the related complications increased. This can be explained by the correct handle of the Schneider membrane perforation [48]. Furthermore, the only implant lost due to non-osseointegration was placed in a maxillary sinus where no membrane perforation was clinically observed. A longer follow-up of this study sample, if patients attend follow-up consultations, will show if membrane perforation can increase the risk of implant failure, as reported in a previous systematic review and meta-analysis [20].

The prosthesis survival rate was excellent. However, one patient presented hardware complications including screws, pilar and ceramic fractures. These complications were very likely associated with the patient’s bruxism condition. Furthermore, despite the advice to use occlusal splints (nightguards), the patient reported not using them. These complications are common and previous studies also reported very similar prosthetic components fractures after loading [44,49], mainly for patients diagnosed with bruxism [50]. Additionally, it is of paramount importance the correct assembly of the prosthesis, including balanced forces distribution, being easy for the patient to maintain it clean through regular hygiene, allowing the periimplant healthy maintenance. The screwed dental prosthesis to the implants was the choice of our patients since excess cement may lead to periimplantitis and consequently to increased bone loss [27].

Commonly used intraoral radiographs to assess marginal bone loss have some limitations because a three-dimensional reality is evaluated in only two dimensions, causing loss of information [32]. However, the cost-benefit for follow-up consultations, no image distortion and low radiation doses make this exam the most used radiographic analysis. Additionally, when the radiographic images are correctly acquired, by employing the paralleling technique, it is possible to evaluate the progression of mean bone loss after implant loading, as demonstrated in the present investigation and other studies [51,52].

Despite the minimized biological variability allowed by the split-mouth design, since each patient acted both as a control and as a test of itself, reducing the needed number of patients in each group [53], there might be a risk that the present study is under-powered. In this context, the limitations in the sample size did not allow further investigation of additional information, such as the influence of smoking habits, age, gender, and systemic diseases. Other limitation of this study is related to the lack of buccal-palatal bone wall distance sinus measurements. It was reported [5], after the surgical procedures performed in the present study, that the buccal-palatal bone wall distance should be considered in treatment planning, since it can influence clinical outcomes.

Nevertheless, this study aimed to test the use of porcine xenograft to overcome some of the limitations of autologous bone. Despite minor statistical differences found, both autologous bone and porcine xenograft are excellent alternatives to allow dental rehabilitation on implants in the posterior maxillae, with no clinical differences for the evaluated patients. Finally, the results of this randomised controlled clinical trial should be interpreted considering the reported limitations.

## 5. Conclusions

The maxillary sinus augmentation procedure is an excellent clinical option procedure for the prosthetic rehabilitation of atrophic maxillae with a high implant survival rate, either by using autologous bone or porcine xenograft materials. Radiographic evaluation of bone loss after one year of loading showed good results for both groups. Particular attention to bruxism and the correct use of occlusal splints are important to avoid prosthetic complications.

## Figures and Tables

**Table 1:** Values of bone lost in millimetres (mm). Comparisons between temporal evaluations of prosthesis delivery (t0) and one year follow-up (t1), and between autologous (A) and xenograft (X) materials, for the combination of Mesial (M) and Distal (D) results, displaying mean, standard-deviation (SD), bootstrapped 95% of confidence intervals (CIs) and *p*-values.

		(M + D)0				(M + D)1				(M + D)1 − (M + D)0				
		Mean	SD	95% CI Lower	95% CI Upper	Mean	SD	95% CI Lower	95% CI Upper	Mean	SD	95% CI Lower	95% CI Upper	*p*-Value
Material	A	0.374	0.510			0.437	0.532			0.063	0.126	0.022	0.105	0.004
	X	0.497	0.614			0.589	0.640			0.092	0.163	0.038	0.146	0.001
	Total	0.436	0.564			0.513	0.590			0.078	0.146			
	Difference (X − A)	0.124		−0.382	0.134	0.153		−0.422	0.117	0.029		−0.096	0.038	
	*p*-value	0.343				0.262				0.390				

**Table 2:** Values of bone lost (mm). Comparisons between temporal evaluations (t0 and t1) and between materials for the mesial (M) and distal (D) groups, displaying mean, SD, bootstrapped 95% CI and *p*-values.

		M0				M1				M1 − M0				
		Mean	SD	95% CI Lower	95% CI Upper	Mean	SD	95% CI Lower	95% CI Upper	Mean	SD	95% CI Lower	95% CI Upper	*p*-Value
Material	A	0.374	0.549			0.432	0.579			0.058	0.122	0.011	0.126	0.129
	X	0.405	0.655			0.484	0.710			0.079	0.165	0.026	0.189	<0.001
	Total	0.389	0.596			0.458	0.640			0.068	0.144			
	Difference (X − A)	0.032		−0.316	0.421	0.053		−0.316	0.468	0.021		−0.063	0.117	
	*p*-value	0.868				0.804				0.625				
		**D0**				**D1**				**D1 − D0**				
		**Mean**	**SD**	**95% CI Lower**	**95% CI Upper**	**Mean**	**SD**	**95% IC Lower**	**95% IC Upper**	**Mean**	**SD**	**95% CI Lower**	**95% CI Upper**	***p*** **-Value**
Material	A	0.374	0.484			0.442	0.497			0.068	0.134	0.026	0.147	<0.001
	X	0.589	0.573			0.695	0.561			0.105	0.165	0.047	0.195	0.008
	Total	0.482	0.535			0.568	0.538			0.087	0.149			
	Difference (X − A)	0.216		−0.105	0.547	0.253		−0.073	0.574	0.037		−0.058	0.132	
	*p*-value	0.222				0.150				0.503				

## Data Availability

Data is available from the corresponding author on reasonable request.

## Abbreviations

RCT	randomised clinical trial
CI	confidence interval
SD	standard deviation
M	mesial
D	distal
A	autologous bone graft
X	porcine xenograft
t0	prosthesis delivery
t1	one year follow-up
mm	millimetres
DBB	demineralized bovine bone
PRP	platelet-rich plasma
